# An overlooked plant–parakeet mutualism counteracts human overharvesting on an endangered tree

**DOI:** 10.1098/rsos.171456

**Published:** 2018-01-31

**Authors:** Karina L. Speziale, Sergio A. Lambertucci, Gabriela Gleiser, José L. Tella, Fernando Hiraldo, Marcelo A. Aizen

**Affiliations:** 1 Grupo de Biología de la Conservación, Laboratorio ECOTONO, INIBIOMA (CONICET-Universidad Nacional del Comahue), Bariloche, Río Negro, Argentina; 2 Grupo de Ecología de la Polinización, Laboratorio ECOTONO, INIBIOMA (CONICET-Universidad Nacional del Comahue), Bariloche, Río Negro, Argentina; 3 Departamento de Biología de la Conservación, Estación Biológica Doñana, CSIC, Sevilla, Spain

**Keywords:** Araucaria, Austral parakeet, monkey puzzle, mutualism, partial consumption, overexploitation

## Abstract

The exponential growth of the human population often causes the overexploitation of resources and disruption of ecological interactions. Here, we propose that the antagonist effect of humans on exploited species might be alleviated with the advent of a second predator species. We focused on the complex interactions between an endangered conifer (*Araucaria araucana*) and two seed exploiters: the Austral parakeet (*Enicognathus ferrugineus*) and human seed collectors. We tested the importance of partial seed consumption by parakeets as an escape from human seed harvesting. Although parakeets frequently ate whole seeds, a substantial proportion of the seeds found under trees were only partially eaten and avoided by human seed collectors. These seeds germinated at a similar proportion but faster than intact seeds under laboratory conditions. Our results revealed an overlooked mutualism between parakeets and an endangered tree. Incomplete seed eating by parakeets, plus selection against these eaten seeds by humans, may enhance regeneration possibilities for this conifer species subject to human seed collection, turning the scale of the antagonism–mutualism continuum to the mutualistic side. In this context, parakeets might be providing an important service in those forests subject to human harvesting by allowing a fraction of seeds to escape human predation.

## 1. Introduction

Biological interactions are far more complex than the interpretations we make from the one-time ‘screen-shots’ we usually use to portray them and the simplistic classification in which we force them. This is even truer when humans come into action. Humans have been part of the interaction networks characterizing most natural ecosystems for thousands of years [1]. However, ever since human populations began to increase exponentially, and especially, since humans started gathering in societies and using new technologies, ecosystem overexploitation and disruption of ecological interactions began [2–4]. In the last decades, the recognition of widespread human–nature interactions and human intrinsic need of intensive resource consumption have changed conservation practices, from the preservation of biodiversity by creating protected areas free of people to the inclusion of the humans in the design of conservation strategies.

Despite these efforts, humans have been proved to be drivers of manifold negative impacts on species interactions worldwide through activities that lead to global change [5]. For instance, habitat fragmentation, a consequence of man-driven land use conversion, disrupts species interactions by altering species and gene movements and differentially contributing to the mortality of interacting species [6]. Global anthropogenic warming and plant invasions break plant–pollinator interactions by altering plant phenology or by introducing new resources for pollinators [7–9]. Also, species overexploitation directly disrupts interactions between species through the differential population decrease of the target species, affecting all the species associations in the ecosystem [10]. In these ways, humans alter species interactions threatening the conservation of whole ecosystem processes and the maintenance of biodiversity [5,11].

The antagonistic effect of humans on exploited species and interactions might be alleviated under a scenario of complex interactions. Complex interactions and their outcomes have been well studied in the case of apparent competition [12–14]. An analogous scenario can occur with predation. Predation has always been considered a negative outcome for one of the interacting species. However, a third party can modify the sign and magnitude of an antagonistic interaction. More specifically, when two predators share a prey species, one of the negative interactions could ameliorate the other negative interaction if non-lethal predation by one predator species reduces the prey likelihood of being chosen by the other predator species. In the case of seed consumers, even sporadic incomplete consumption of single seeds may be advantageous for a plant, when partially eaten seeds retain at least some germination potential while they become less attractive for other seed predators. Large seeds may be more resistant to damage by seed consumers as they may retain germination potential after being damaged [15]. Thus, partial consumption of seeds from a large-seeded species may help to reduce further seed consumption by a second predator species, which could lead to an increase in seed dispersal and germination potentials.

One of the most evident interactions in the monkey puzzle (*Araucaria araucana* (Mol) K. Koch) forests of southern South America occurs between this charismatic conifer and the Austral parakeet (*Enicognathus ferrugineus*). The globally endangered *A. araucana* tree occupies a restricted range across the eastern (Argentina) and western (Chile) slopes of the northern Patagonian Andes [16]. The geographic range of these forests is considered among the 200 areas of the world with the highest conservation interest [17]. These temperate forests have historically been affected by fires, overgrazing, wood exploitations and, more recently, by land fragmentation. These threats lead to a current distribution area of only around 400 km^2^ out of a potential past distribution of 5000 km^2^ [18] with the most obvious sign of forest degradation being the lack of natural regeneration [19]. The Austral parakeet concentrates in *A. araucana* forests during autumn–winter, when it mostly forages on *A. araucana* seeds, thus contributing to their secondary dispersal after being primarily dispersed by barochory. A recent study showed that at least 57% of the seeds produced are moved by Austral parakeets up to 50 m from the maternal tree to be consumed in distant perch trees. Whereas this parakeet eats most seeds completely, some of them are dropped without damage when they are handled for consumption, thus providing a mutualist service [20], and others are eaten incompletely. This opens the question that at least some of these incompletely consumed seeds could retain germination potential (given that *A. araucana* is a large-seeded species), as was recently shown for the sister species *Araucaria angustifolia* [20]. To increase complexity, humans also harvest and consume *A. araucana's* seeds, and thus can play an important role, at least in relative terms, in determining the outcome of the interaction between the endangered tree and the parakeet.

Humans represent a direct threat as seed consumers, and an indirect threat through the introduction of other seed predators into *A. araucana* forests such as cattle, wild boars and deer that feed on *A. araucana* seeds [21,22]. Both processes result in a reduction in the availability of *A. araucana* seeds for their consumption and dispersal by Austral parakeet and for *A. araucana* forest regeneration [21]. The traditional consumption of seeds by original human gatherers changed drastically as collection extended to other social groups and with the onset of a commercial trade of *A. araucana* seeds [23]. Though the impact of human collection has not been properly measured across the whole *A. araucana* distribution range, this activity has been recognized as an important threat impairing forest regeneration, particularly for the tree populations most accessible to humans [24]. *A. araucana* is listed as an Endangered species by the IUCN Red List and its international trade is not allowed as it was listed on Appendix I of CITES [16]. Despite this status, human collection takes place every autumn, the period of maximum seed production and dispersal. Collection is allowed by the Argentinian government, which yearly regulates collection quotas and areas allowed for extraction, although with almost null capacity to enforce these measures. In 2016, by resolution 042/16, the government of Neuquén province in Argentina allowed a collection rate up to 20 kg/person and 100 kg/family for personal consumption after obtaining a permit that cost 0.35 US$/kg. Collection for commercialization was taxed at 0.7 US$/kg and each person allowed to harvest up to 300 kg. Worryingly, there were neither restrictions in the number of permits sold nor studies on sustainable extraction rates.

The multiple ecological interactions involving *A. araucana* trees, parakeets and humans constitute an excellent system to address whether partial seed damage might constitute an escape from human harvesting. This will be the case if humans avoid collecting seeds partially damaged by parakeets, provided these seeds retain germination potential. In our study system, the antagonistic–mutualistic continuum between Austral parakeets and *A. araucana* trees could move towards mutualism in the presence of human predation, constituting an overlooked case of plant–animal mutualism. So, focusing solely on the antagonistic aspect *A. araucana*–parakeet seed predation interaction, we ask whether seed partially consumed by parakeets could increase the potential fitness of female trees by reducing the amount of seeds removed by humans while not compromising the germination potential of seeds. Human seed collection may impact female fitness negatively because it reduces potential regeneration under seed-producing trees [20,25,26] and the seed pool available for secondary dispersal by Austral parakeets and mice [20,27]. We hypothesized that seeds damaged by Austral parakeets are not attractive for humans and thus they escape from anthropic seed collection, increasing the amount of seeds available for *in situ* germination and secondary dispersal despite human seed exploitation pressure. Based on this hypothesis, we predicted that (i) humans will collect intact seeds; (ii) *A. araucana* trees subjected to human collection will show much lower density of intact seeds underneath than trees not subject to human seed collection; (iii) seeds damaged by Austral parakeets will retain germination potential, (iv) the presence of seeds partially damaged by parakeets will increase seed dispersal efficiency (SDE) and (v) this increment in SDE will be more important in those areas subject to human seed collection. Results supporting these predictions would demonstrate that in the presence of humans, parakeet seed predation behaviour can increase recruitment potential of an endangered and emblematic tree.

## 2. Material and methods

### 2.1. Study area and species

**Figure 1.**
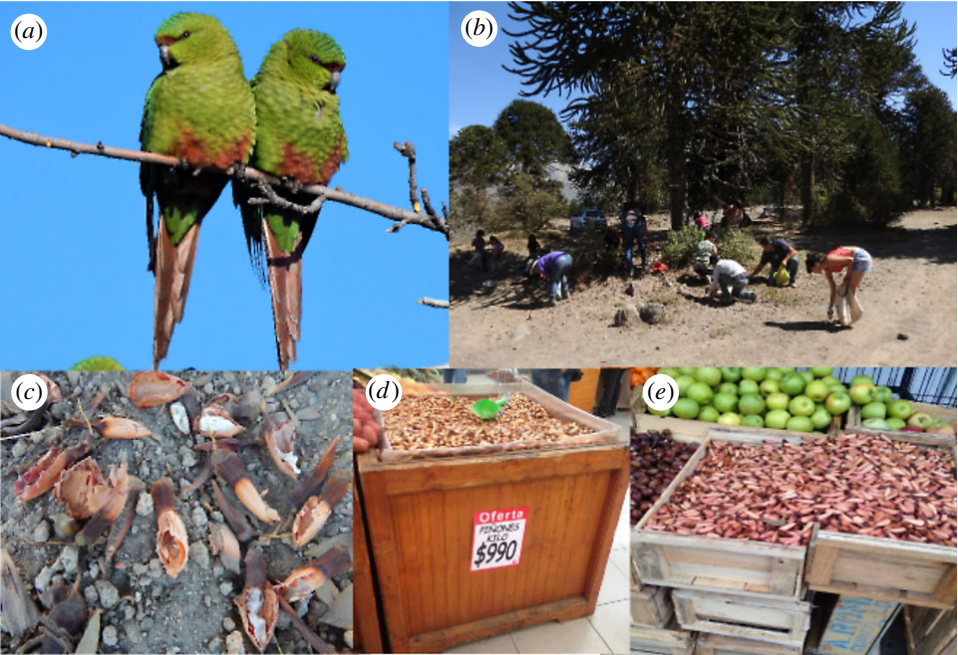
(*a*) Austral parakeets perching on *Araucaria araucana*. (*b*) Group of gatherers collecting seeds. (*c*) Seeds commonly found on the ground, intact, partially eaten and completely eaten by parakeets. *Araucaria araucana* seeds sold in markets in (*d*) Chile and (*e*) Argentina.

The native geographic range of *A. araucana* is located in the south of Argentina and Chile and comprises two separate areas, the Andes Cordillera (37°30′ to 40°02′ S) and the coastal Cordillera of Nahuelbuta (between 37°20′ and 38°40′ S) [28]. Our study was conducted in Lanin National Park, Argentina (39°12'37′′ to 39°14'27′′ S and 71°09'15′′ to 71°9'35′′ W and close surroundings). The *A. araucana* tree (‘monkey puzzle’ in English, ‘Araucaria’ in Spanish and ‘Pehuen’ in Mapudungun), an endangered and highly endemic conifer tree [16,29], is dioecious (rarely monoecious) and reaches sexual maturity after 20–30 years [24]. It is a masting species with highly variable seed production among years, most likely a strategy to satiate predators [30]. Female cones produce between 100 and 200 seeds [24,30], each weighing 3.5 g, on average (figure 1) [30]. Seeds are dispersed by barochory; they are dispersed naturally or when they fall as Austral parakeets feed on female cones, falling under or a few metres away from the seed-producing tree. Seed ripening occurs from February to May, peaking in April [18]. Long- distance dispersal is performed by Austral parakeets up to at least 50 m, but most probably to longer distances [21] and rodents up to 40 m [27]. However, among rodents, only one mice species (*Abrothrix longipilis)* deposits seeds in potential good places for germination. Nevertheless, germination normally occurs when seeds are on top soil while this rodent buries them under soil or leaf litter between 7 and 9.4 cm deep [27].

The seeds are rich in carbohydrates, particularly starch [31], and germinate soon after dispersal, which coincides with the onset of the rainy season [32]. Thus, the large, recalcitrant seeds of *A. araucana* do not form a persistent seed bank and recruitment depends on the number of seeds produced and surviving each year [33]. *A. araucana* is shade tolerant when young, germinating in any type of microhabitat, including the ground under the maternal tree [18,25,34,35]. In fact, in a recent study we recorded a total of 3305 saplings and 550 young trees growing under the putative parental trees, when carefully inspecting under the canopy of 516 female *A. araucana* trees [21].

*A. araucana* seeds have traditionally constituted an important source of carbohydrates for the Pehuenche/Mapuche people living in the south of Argentina and Chile [22]. Seeds are consumed in many different ways: raw, boiled, toasted, ground as flour and as an alcoholic beverage called ‘mudai’ [28,36]. Moreover, this staple is used to feed cattle particularly during winter time [22,28]. In addition to native seed predators (including Austral parakeets, native rodents and some invertebrates), several introduced, non-native vertebrates besides men (e.g. wild boar, rabbit, red deer, European hare, livestock) consume *A. araucana* seeds [20,31,37].

In the study area, humans used three different techniques to collect seeds not dispersed naturally: (i) hitting female cones with long sticks, a technique that only allowed access to the lower cones; (ii) using ropes for accessing some higher female cones; (iii) climbing up to the top of female trees and hitting cones, which was the most effective and destructive method.

### 2.2. Field sampling

We visited four *A. araucana* forest patches (=stands) in the Tromen area of Lanin National Park (*ca* 39°36′52′′ S, 71°20′59′′ W) in northwestern Argentinian Patagonia at the peak of the seed dispersal period in autumn 2016. Even though the selected study area is not listed among those allowed for collection, it was interesting to test our hypothesis in an area that was subjected to human overharvesting within a National Park in which seed collection occurs intensively regardless of being forbidden. Three of these patches were subject to anthropogenic seed collection, while the remaining one, experiencing no collection because of being close to the park ranger station and customs office, was used as control. At each area, whenever we saw people collecting *A. araucana* seeds, we asked for permission to randomly inspect 50 of the seeds collected in their bags (*N* = 10), and we recorded the number of entire seeds and of those partially eaten by parakeets (with part of the seed lost due to the bite of a parakeet). We asked collectors that accepted to be interviewed whether they selected seeds based on any characteristic (*N* = 10 groups of people). Additionally, we randomly collected 30 seeds from the ground under each tree (the number of collected seeds was limited by the small number of seeds remaining underneath trees) where humans have been previously collecting, recording also the number of entire seeds and of those partially damaged by parakeets.

Additionally, we estimated seed density by sampling seeds underneath the crown of a total of 50 seed-producing *A. araucana* trees right after the seed collectors left the tree (20 trees in each of two of the patches and 10 trees in the third one). We also sampled seeds under the canopy of 10 trees in the control area. We estimated seed density by haphazardly throwing 10 times a 40 × 50 cm frame within the area projected by the crown of each of the sampled trees, and counting each time the number of intact and partially damaged seeds within the area delimited by the frame. Finally, under each tree we collected intact and partially eaten seeds, which we stored for later use in the germination laboratory experiment.

### 2.3. Germination trials

We assessed the germination potential of seeds partially eaten by parakeets under laboratory conditions. We considered as damaged seeds those with less than 50% of the seed consumed. We calculated the germination rate of three different seed types (with 50 replicates each): (i) intact seeds, (ii) seeds partially eaten by parakeets, and (iii) seeds in which we simulated the effect of parakeet damage by manually cutting the distal portion of the seed in similar proportions as those munched by parakeets. This third treatment was included to assess whether parakeet predation affects seed germination beyond any effect related to the partial removal of tegument and endosperm. We randomly selected the seeds from the seed pool collected under 22 of the sampled trees. We weighed and measured the length of each seed, and then placed them individually in a 10 × 15 cm plastic tray on a layer of moist paper towel contained in a 10 × 15 cm plastic tray. The tray was then sealed with a plastic film to avoid dehydration. Trays were randomly mixed and incubated in a germination chamber set at 23°C and with a cycle of 14/10 light/dark hours, as recommended by Duplancic [38]. Seeds were treated with Fungoxan (Carbendazim, 1 ml l^−1^), a common procedure in germination experiments to avoid seed moulding, and paper towels changed regularly [38]. We inspected seeds twice a week (i.e. each 3–4 days) during 57 days after the start of the germination trials, recording the number of seeds that showed a protruded radicle.

### 2.4. Data analysis

Data (electronic supplementary material) were analysed using generalized linear mixed effects models (GLMMs). First, we assessed whether the proportion of partially eaten seeds differed between seed pools collected from people's bags and those collected underneath trees considering a logit-link function and a Binomial error distribution, and including ‘group’ (i.e. gatherer or tree) as a random term. Second, we assessed whether the number of intact seeds differed between trees in areas free and not free from human seed harvesting using a log-link function and a Poisson error distribution. Third, we assessed whether seed germination (coded as a binary variable) differed among treatments (i.e. intact, partially consumed predated by parakeets and hand-cut) at the end of the trial, using a GLMM with logit-link function and Binomial error distribution, including seed weight and seed length as covariates and ‘tree’ as a random term to account for any potential maternal effect in seed quality. In a fourth analysis, we used a GLMM with a similar structure as the previous analysis, but with a Gamma error distribution, to analyse whether the germination speed (number of days that elapsed until each seed germinated) differed among the three treatments. This analysis was restricted to those seeds that germinated. All analyses were performed using the package ‘lme4’ [39] in R [40].

We then compared *A. araucana* seed dispersal efficiency (SDE) [41] to provide estimates of seedling recruitment in four scenarios: (i) intact seeds in control area (without human seed collection), (ii) partially consumed seeds in control area, (iii) intact seeds in areas subject to human collection and (iv) partially consumed seeds plus intact seeds in areas subject to human collection. The analysis of SDE is a good approach to evaluate dispersal success [42]. We measured this SDE index only for the seeds primarily dispersed by barochory or by parakeets under maternal trees. Given that we did not quantify the proportion of germinated seeds turning into saplings we could only estimate SDE by considering germination potential of primarily dispersed seeds (i.e. not considering long-distance dispersal by parakeets [21]). As a consequence, this index was estimated by multiplying the median seed density found under trees in each scenario (a quantitative component of dispersal) by the germination potential of intact or partially consumed seeds obtained from our previous results (a qualitative component of dispersal [43]). We expected that SDE of partially consumed seeds will not contribute to increase total SDE significantly in control areas. On the other hand, its contribution to total seedling recruitment would be significant in areas subject to human seed collection given that SDE of intact seeds in this area would be very low.

## 3. Results

**Figure 2.**
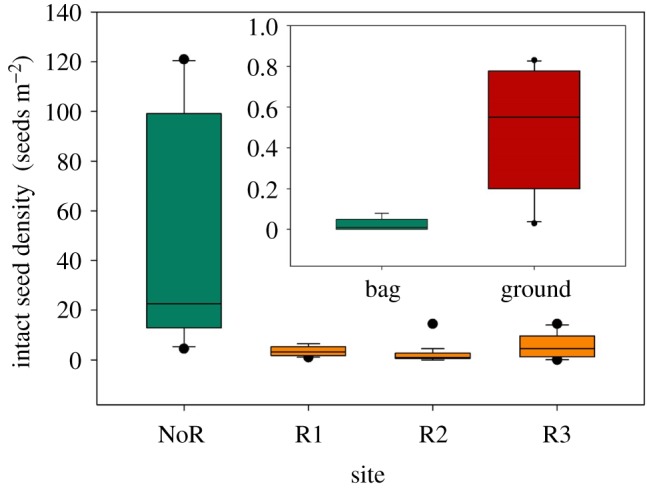
Differences in intact seed density among the four study areas. R1 (*N* = 20), R2 (*N* = 20) and R3 (*N* = 10): three sites with human collection; NoR: site without human collection (*N* = 10). *N* = number of trees sampled in each site. Inset: box plot showing partially eaten seed proportion (eaten/intact seed pools) measured on the ground under *Araucaria araucana* trees subject to human collection and inside gatherers' bags. Both box plots show median values (central horizontal lines), 25th and 75th percentile regions around the median value (box limits), 10th and 90th percentiles (whiskers) and outliers (circles).

A small number of illegal collectors accepted to be interviewed (*N* = 10 groups); all of them stated that they avoided collecting partially eaten seeds. Accordingly, the mean proportion of partially eaten seeds in people's bags was 2% (range= 0–8%), whereas the mean proportion of partially eaten seeds below trees where people collected seeds was 49% (range= 6.7–83.3%; *z* = 11.88; *p* < 0.001; figure 2, inset). Because of human gathering behaviour, the density of intact seeds was much lower underneath trees subject to human collection (median 2 seeds m^−2^; range 0–14.5 seeds m^−2^, average 3.24 seeds m^−2^) than underneath trees without collection (median 21 seeds m^−2^; range 4–121 seeds m^−2^; average 42.5 seeds m^−2^, figure 2) (z = 7.14, *p *< 0.001). The density of partially damaged seeds underneath trees subject to human collection was similar to that of intact seeds (median 1 partially damaged seeds m^−2^, range 0–27 seeds m^−2^, average 2.47 seeds m^−2^).

**Figure 3.**
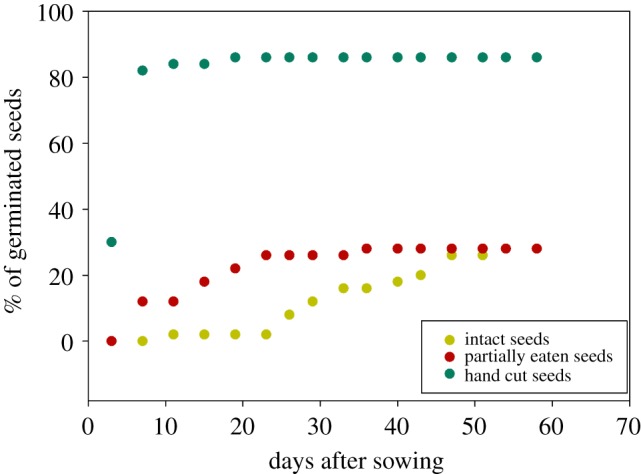
Proportion of germinated seeds in relation to days passed after sowing for three treatments. Total seeds in each treatment = 50.

The experimental trials showed that germination strongly differed among seed types (figure 3). The greatest germination success (86% of seeds) was obtained from those seeds that were hand-cut before being sown (table 1 and figure 3). Hand-cut seeds germinated faster than partially eaten seeds, whereas intact seeds germinated at the lowest rate. However, germination success was similar for both intact and partially eaten seeds (28% of germinated seeds for both treatments). Seed germination was neither affected by seed weight nor by length in any of the three treatments after controlling for maternal tree (table 1 and figure 3).

**Table 1.** Generalized linear mixed models explaining differences in: (*a*) germination success (yes/no); (*b*) germination speed (number of days elapsed until germination).

fixed effects	estimate	s.e.	*z*-value	*p*
(*a*) germination yes/no				
(intercept)	−1.60	1.85	−0.81	0.389
weight	0.51	0.34	1.41	0.149
length	−0.02	0.05	−0.48	0.633
hand-cut	2.36	0.50	4.68	<0.001
partially predated	−0.42	0.53	−0.80	0.422
(*b*) germination speed				
(intercept)	2.05	0.65	3.13	0.001
weight	0.05	0.10	0.48	0.63
length	0.004	0.02	0.26	0.80
hand-cut	−1.79	0.16	−11.51	<0.001
partially predated	−1.02	0.18	−5.68	<0.001

Given the median seeds m^−2^ and germination success shown above, the SDE was 5.88 seedlings m^−2^ in control areas, 0.56 seedlings m^−2^ in control areas for partially consumed seeds, 0.7 seedlings m^−2^ for intact seeds in areas subject to human collection, and 1.26 seedlings m^−2^ when considering the combined effect of intact and partially damaged seeds in areas subject to human collection. Thus, in areas with high seed collection by humans, SDE was almost 80% higher when partially damaged seeds are included.

## 4. Discussion

Human overexploitation of natural resources can disrupt interactions and drive species to extinction [44,45]. However, here we show how interactions with third parties can buffer human impact. Because people avoided harvesting seeds munched by parakeets, incomplete seed consumption could represent an important escape from human predation for *A. araucana* seeds. As damaged seeds germinated in similar proportions as intact seeds, the escape from human exploitation promoted by parakeets' predation behaviour could become critical for maintaining the population viability of *A. araucana* forests, particularly in those areas subject to anthropic seed collection. In areas under heavy harvesting by humans, the increment of almost 80% in SDE of seeds partially consumed by parakeets could promote a significant demographic effect given that an increment in recruitment at low seed densities could disproportionally improve growth rate in long-lived species. Most seeds produced during inter-mast years are expected to be lost due to predation by parakeets. These birds predate completely up to 20% of the seeds produced by a tree, and 52% of seeds are greater than 50% damaged [37]. Thus, parakeets have been considered as antagonistic interactors until their mutualistic role as dispersers was recently discovered [20] and their role as pollinators was suggested [46]. Moreover, parakeet feeding behaviour (i.e. partially eating and discarding many of the seeds, up to 40% in masting years [37]) plus the avoidance of collection by humans of partially eaten seeds may enhance *A. araucana* regeneration. This increases the mutualistic component in the antagonistic–mutualistic continuum of a plant–bird interaction.

The partial removal of the coat (and of part of the embryo) by parakeets enhanced the germination speed of *A. araucana* seeds. Seed coat removal by frugivores has also been observed in other plant species manipulated by primates and macaws, which scratch, scarify or remove parts of the fruit and often drop seeds or fruit during manipulation [47]. For instance, in forests of *Araucaria angustifolia*, in Brazil, a high proportion of seeds that were damaged by several parakeet and parrot species [20] germinated in the field, suggesting that partial consumption is a common feeding behaviour among parrots and that in many instances is a non-lethal seed predation behaviour [48–50]. The germination success of hand-cut seeds was greater than that of those partially damaged by parakeets (figure 3); this could be explained by the time at which seed pericarp was partially removed in each case. Pericarps from hand-cut seeds were removed just before the start of the *in vitro* experiment, whereas partially eaten seeds have remained as such in the field for variable time periods, being probably exposed, on average, for longer times to fungus, dehydration or other factors negatively affecting germination [38]. Also, germination success of hand-cut seeds was greater than that of intact seeds, demonstrating that partial removal of the seed coat increases germination success. Apart from not affecting germination potential, partial consumption of seeds increased germination speed and the time window for germination as damaged seeds started germinating much earlier. This could increase the chances of recruitment through limiting the time window for seed predation by terrestrial vertebrates (e.g. wild boars, rodents, etc.) and environmental stress, reinforcing the idea that the interaction between *A. araucana* trees and Austral parakeets has a novel mutualistic component not recognized till now.

The importance of this parakeet–*A. araucana* mutualism seems to be widespread across *A. araucana*'s geographical range, as signs of parakeet feeding were recorded in 85% out of 516 trees distributed throughout this tree range distribution, whereas human seed collection was recorded in 10 out of 24 areas surveyed in both Chile and Argentina ([21]; authors' unpublished data). During the annual *A. araucana* seed harvesting period, families can collect up to 2000–3000 kg of seeds [51]. Mapuche-Pewenche people might have acted as seed dispersers while moving them across the landscape [52]. However, some groups of Mapuche-Pewenche people changed their interaction with *A. araucana* forests due to recent cultural changes [51]. Additionally, new groups of city-dweller collectors visit the area during weekends to collect seeds. The fact that current seed collection depletes seed availability represents an excessive cost for *A. araucana* trees, Austral parakeets, and for the whole assemblage of native seed predators. However, the fact that collectors are selective against damaged seeds turns partial seed consumption by parakeets into an effective mechanism for this tree to escape human predation. Thus, the persistence of partially eaten seeds on the ground is relevant as it reduces human negative impact and promotes germination under or close to the parental tree [24].

Until recently, the antagonistic role of parakeets and parrots in general as seed predators has been emphasized in detriment of the recognition of other plant–parrot mutualisms [20,21,53]. In particular, the germination potential of partially eaten seeds has been rarely addressed [15,47,54,55], and, to our knowledge, partial seed predation has never been considered as a possible mechanism rescuing wild plant populations subjected to human exploitation. Thus, along the continuum antagonism–mutualism, partial seed consumption turns the outcome of the interaction towards a more positive result. In this context, our study suggests that conservation of the anthropogenic-threatened *A. araucana* forests could be favoured by Austral parakeet partial seed consumption, particularly, in the presence of human overharvesting. However, forest conservation in human-impacted areas cannot only rely on the feeding behaviour of an endemic parakeet. Fostering education and human behavioural change to reduce the number of illegal harvesters, particularly in protected areas, and enforcement of conservation laws are needed. Restoration initiatives are already taking place in Chile, where Mapuche communities are planting seeds and nursing seedlings [28]. Also, the study and monitoring of the sustainability of human collection merit special attention, to better predict its impact on Araucaria forests regeneration potential and to provide scientific means to regulate harvesting as it is suggested for other tree species in South America [26]. In any event, the interaction between *A. araucana* and parakeets we describe here could be a key element in developing a realistic conservation strategy focused on these emblematic forests.

Human seed collection, a selection mechanism directed particularly towards perfect and intact items, is a widespread phenomenon [56,57], common even since prehistorical times [58]. In fact, escape from human harvesting could also occur in other regions of the world where wild seeds and fruits are collected by human inhabitants. Thus, partial seed consumption by wild frugivores when seeds retain germination potential, increase total SDE, and could favour ecosystems survival when subject to human intense harvesting. Both behaviours, partial seed damage by frugivorous species together with selection against predated fruits or seeds by humans, may enhance regeneration possibilities for many plant species worldwide, having relevance at a global scale beyond our study system.

## Supplementary Material

Proportion of partially eaten seeds between people's bags and those collected underneath trees;Intact seed density comparison;Influence of seed size and weight on the germination results;Proportion of germinated seeds in relation to days passed after sowing

## Data accessibility

Data are available to download from Dryad Digital Repository: http://dx.doi.org/10.5061/dryad.9g263 [59].

## References

[1] Holt FL 2005 The catch-22 of conservation: indigenous peoples, biologists, and cultural change. Hum. Ecol. 33, 199–215. (doi:10.1007/s10745-005-2432-X)

[2] Stahl PW 1996 Holocene biodiversity: an archaeological perspective from the Americas. Annu. Rev. Anthropol. 25, 105–126. (doi:10.1146/annurev.anthro.25.1.105)

[3] Reynolds JD, Mace GM, Redford KH, Robinson JG (eds) 2001 Conservation of exploited species. Cambridge, MA: Cambridge University Press.

[4] Hames R 2007 The ecologically noble savage debate. Annu. Rev. Anthropol. 36, 177–190. (doi:10.1146/annurev.anthro.35.081705.123321)

[5] Chapin FS III et al. 2000 Consequences of changing biodiversity. Nature 405, 234–242. (doi:10.1038/35012241) 10821284 10.1038/35012241

[6] Fagan WF, Cantrell RS, Cosner C 1999 How habitat edges change species interactions. Am. Nat. 153, 165–182. (doi:10.1086/303162) 10.1086/303162 29578760

[7] Memmott J, Craze PG, Waser NM, Price MV 2007 Global warming and the disruption of plant–pollinator interactions. Ecol. Lett. 10, 710–717. (doi:10.1111/j.1461-0248.2007.01061.x) 17594426 10.1111/j.1461-0248.2007.01061.x

[8] Aizen MA, Morales CL, Morales JM 2008 Invasive mutualists erode native pollination webs. PLoS Biol. 6, e31 (doi:10.1371/journal.pbio.0060031) 18271628 10.1371/journal.pbio.0060031 PMC2235906

[9] Traill LW, Lim ML, Sodhi NS, Bradshaw CJ 2010 Mechanisms driving change: altered species interactions and ecosystem function through global warming. J. Anim. Ecol. 79, 937–947. (doi:10.1111/j.1365-2656.2010.01695.x) 20487086 10.1111/j.1365-2656.2010.01695.x

[10] Estes JA, Palmisano JF 1974 Sea otters: their role in structuring nearshore communities. Science 185, 1058–1060. (doi:10.1126/science.185.4156.1058) 17738247 10.1126/science.185.4156.1058

[11] Bascompte J, Jordano P, Olesen JM 2006 Asymmetric coevolutionary networks facilitate biodiversity maintenance. Science 312, 431–433. (doi:10.1126/science.1123412) 16627742 10.1126/science.1123412

[12] Holt RD 1977 Predation, apparent competition, and the structure of prey communities. Theor. Popul. Biol. 12, 197–229. (doi:10.1016/0040-5809(77)90042-9) 929457 10.1016/0040-5809(77)90042-9

[13] Chaneton EJ, Bonsall MB 2000 Enemy-mediated apparent competition: empirical patterns and the evidence. Oikos 88, 380–394. (doi:10.1034/j.1600-0706.2000.880217.x)

[14] Chase JM, Abrams PA, Grover JP, Diehl S, Chesson P, Holt RD, Richards SA, Nisbet RM, Case TJ 2002 The interaction between predation and competition: a review and synthesis. Ecol. Lett. 5, 302–315. (doi:10.1046/j.1461-0248.2002.00315.x)

[15] Mack AL 1998 An advantage of large seed size: tolerating rather than succumbing to seed predators. Biotropica 30, 604–608. (doi:10.1111/j.1744-7429.1998.tb00100.x)

[16] IUCN. 2015 The IUCN Red List of Threatened Species. Version 2015.1.

[17] Olson DM, Dinerstein E 2002 The global 200: priority ecoregions for global conservation. Ann. Mo. Bot. Gard. 89, 199–224. (doi:10.2307/3298564)

[18] Donoso C 2006 Las especies arbóreas de los bosques templados de Chile y Argentina: autoecología, 1st edn Valdivia, Chile: Marisa Cuneo Ediciones.

[19] Gallo L et al. 2004 Los recursos genéticos silvícolas de Araucaria araucana en Argentina. In *Desafíos Ord. Los Recur. Genéticos Silvícolas Para Contrib. Subsist. Ej. Argent. Braz* Rome, Italy: IPGRI.

[20] Tella JL, Dénes FV, Zulian V, Prestes NP, Martínez J, Blanco G, Hiraldo F 2016 Endangered plant-parrot mutualisms: seed tolerance to predation makes parrots pervasive dispersers of the Parana pine. Sci. Rep. 6, 31709 (doi:10.1038/srep31709) 27546381 10.1038/srep31709 PMC4992845

[21] Tella JL, Lambertucci SA, Speziale KL, Hiraldo F 2016 Large-scale impacts of multiple co-occurring invaders on monkey puzzle forest regeneration, native seed predators and their ecological interactions. Glob. Ecol. Conserv. 6, 1–15. (doi:10.1016/j.gecco.2016.01.001)

[22] Aagesen DL 1998 Indigenous resource rights and conservation of the monkey-puzzle tree (*Araucaria araucana*, Araucariaceae): a case study from southern Chile. Econ. Bot. 52, 146–160. (doi:10.1007/BF02861203)

[23] Donoso S, Peña-Rojas K, Pacheco C, Perry F, Espinoza C, Pintana S 2010 Evolución de la sustentabilidad de los bosques de *Araucaria araucana*: producción, colecta y consumo de piñones. Span. J. Rural Dev. 1, 99–112. (doi:10.5261/2010.GEN2.08)

[24] Muñoz R 1984 Análisis de la productividad de semillas de *Araucaria araucana* (Mol.) C. Koch en el área de Lonquimay-IX Región. Tesis para optar al Titulo de Ingeniero Forestal, Chile.

[25] Sanguinetti J, Kitzberger T 2009 *Araucaria araucana* temporal and spatial seedling establishment patterns: masting, seed predation and understory vegetation effects. Rev. Chil. Hist. Nat. 82, 319–335. (doi:10.4067/S0716-078X2009000300001)

[26] Peres CA et al. 2003 Demographic threats to the sustainability of Brazil nut exploitation. Science 302, 2112–2114. (doi:10.1126/science.1091698) 14684819 10.1126/science.1091698

[27] Shepherd JD, Ditgen RS 2012 Rodent handling of *Araucaria araucana* seeds. Austral Ecol. 38, 23–32. (doi:10.1111/j.1442-9993.2012.02366.x)

[28] Herrmann TM 2005 Knowledge, values, uses and management of the *Araucaria araucana* forest by the indigenous Mapuche Pewenche people: a basis for collaborative natural resource management in southern Chile. Nat. Resources Forum 29, 120–134.

[29] Hoffmann A 1991 *Flora silvestre de Chile. Zona araucana*, 2nd edn. Santiago, Chile: Ed. Claudio Gay.

[30] Sanguinetti J, Kitzberger T 2008 Patterns and mechanisms of masting in the large-seeded southern hemisphere conifer *Araucaria araucana*. Austral Ecol. 33, 78–87. (doi:10.1111/j.1442-9993.2007.01792.x)

[31] Díaz S 2012 Aspectos dietarios, reproductivos y de preferencia de hábitat de Enicognathus ferrugineus (Aves, Psittacidae) en bosques de *Araucaria araucana*. PhD thesis, Universidad Nacional del Comahue, Bariloche, Argentina.

[32] Paruelo JM, Beltran A, Jobbagy E, Sala OE, Golluscio RA 1998 The climate of Patagonia: general patterns and controls on biotic processes. Ecol. Austral 8, 85–101.

[33] Farnsworth E 2000 The ecology and physiology of viviparous and recalcitrant seeds. Annu. Rev. Ecol. Syst. 31, 107–138. (doi:10.1146/annurev.ecolsys.31.1.107)

[34] Burns BR 1991 The regeneration dynamics of *Araucaria araucana*. PhD thesis, Boulder, CO: Colorado University.

[35] Armesto JJ, villagrán C, Arroyo MTK 1996 *Ecología de los bosques nativos de Chile*. 1st edn. Santiago de Chile, Chile: Editorial Universitaria.

[36] Cardemil L, Reinero A 1982 Changes of *Araucaria araucana* seed reserves during germination and early seedling growth. Can. J. Bot. 60, 1629–1638. (doi:10.1139/b82-211)

[37] Shepherd JD, Ditgen RS, Sanguinetti J 2008 *Araucaria araucana* and the Austral parakeet: pre-dispersal seed predation on a masting species. Rev. Chil. Hist. Nat. 81, 395–401.

[38] Duplancic MA, Martinez Carretero E, Cavagnaro B, Herrera Moratta M, Navas Romero AL 2015 Factors affecting germination of *Araucaria araucana* (*Araucariaceae*) seeds from the xeric forest. Rev. Fac. Cienc. Agrar. Univ. Nac. Cuyo 47, 71–82.

[39] Bates D, Mächler M, Bolker B, Walker S 2014 Fitting linear mixed-effects models using lme4. *ArXiv Prepr. ArXiv14065823*

[40] R Core Team. 2016 *R: A language and environment for statistical computing*. Vienna, Austria: R Foundation for Statistical Computing. See https://www.R-project.org/.

[41] Camargo PH, Martins MM, Feitosa RM, Christianini AV 2016 Bird and ant synergy increases the seed dispersal effectiveness of an ornithochoric shrub. Oecologia 181, 507–518. (doi:10.1007/s00442-016-3571-z) 26899481 10.1007/s00442-016-3571-z

[42] Schupp EW, Jordano P, Gómez JM 2010 Seed dispersal effectiveness revisited: a conceptual review. New Phytol. 188, 333–353. (doi:10.1111/j.1469-8137.2010.03402.x) 20673283 10.1111/j.1469-8137.2010.03402.x

[43] Culot L, Huynen M-C, Heymann EW 2015 Partitioning the relative contribution of one-phase and two-phase seed dispersal when evaluating seed dispersal effectiveness. Methods Ecol. Evol. 6, 178–186. (doi:10.1111/2041-210X.12317)

[44] Murali KS, Shankar U, Shaanker RU, Ganeshaiah KN, Bawa KS 1996 Extraction of non-timber forest products in the forests of Biligiri Rangan Hills, India. 2. Impact of NTFP extraction on regeneration, population structure, and species composition. Econ. Bot. 50, 252–269. (doi:10.1007/BF02907329)

[45] Morris RJ 2010 Anthropogenic impacts on tropical forest biodiversity: a network structure and ecosystem functioning perspective. Phil. Trans. R. Soc. B 365, 3709–3718. (doi:10.1098/rstb.2010.0273) 20980318 10.1098/rstb.2010.0273 PMC2982004

[46] Gleiser G, Lambertucci SA, Speziale KL, Hiraldo F, Tella JL, Aizen MA 2017 The southernmost parakeet might be enhancing pollination of a dioecious conifer. Ecology 98, 2969–2971. (doi:10.1002/ecy.1938) 28846799 10.1002/ecy.1938

[47] Norconk MA, Grafton BW, Conklin-Brittain NL 1998 Seed dispersal by neotropical seed predators. Am. J. Primatol. 45, 103–126. (doi:10.1002/(SICI)1098-2345(1998)45:1<103::AID-AJP8>3.0.CO;2-#) 9573445 10.1002/(SICI)1098-2345(1998)45:1<103::AID-AJP8>3.0.CO;2-#

[48] Blanco G, Hiraldo F, Tella JL 2018 Ecological functions of parrots: an integrative perspective from plant life cycle to ecosystem functioning. Emu-Austral Ornithol. 118, 1–14.

[49] Baños-Villalba A, Blanco G, Díaz-Luque JA, Dénes FV, Hiraldo F, Tella JL 2017 Seed dispersal by macaws shapes the landscape of an Amazonian ecosystem. Sci. Rep. 7, 7373 (doi:10.1038/s41598-017-07697-5) 28785083 10.1038/s41598-017-07697-5 PMC5547140

[50] Montesinos-Navarro A, Hiraldo F, Tella JL, Blanco G 2017 Network structure embracing mutualism–antagonism continuums increases community robustness. Nat. Ecol. Evol. 1, 1661 (doi:10.1038/s41559-017-0320-6) 28970589 10.1038/s41559-017-0320-6

[51] Herrmann TM 2006 Indigenous knowledge and management of *Araucaria araucana* forest in the Chilean Andes: implications for native forest conservation. Biodivers. Conserv. 15, 647–662. (doi:10.1007/s10531-005-2092-6)

[52] Reis M, Ladio A, Peroni N 2014 Landscapes with Araucaria in South America: evidence for a cultural dimension. Ecol. Soc. 19, 43–56. (doi:10.5751/ES-06163-190243)

[53] Blanco G, Bravo C, Pacifico EC, Chamorro D, Speziale KL, Lambertucci SA, Hiraldo F, Tella JL 2016 Internal seed dispersal by parrots: an overview of a neglected mutualism. PeerJ 4, e1688 (doi:10.7717/peerj.1688) 26925322 10.7717/peerj.1688 PMC4768710

[54] Manzur MI, Courtney SP 1984 Influence of insect damage in fruits of hawthorn on bird foraging and seed dispersal. Oikos 43, 265–270. (doi:10.2307/3544142)

[55] Perea R, San Miguel A, Gil L 2011 Leftovers in seed dispersal: ecological implications of partial seed consumption for oak regeneration. J. Ecol. 99, 194–201. (doi:10.1111/j.1365-2745.2010.01749.x)

[56] Castle LM, Leopold S, Craft R, Kindscher K 2014 Ranking tool created for medicinal plants at risk of being overharvested in the wild. Ethnobiol. Lett. 5, 77–88. (doi:10.14237/ebl.5.2014.169)

[57] Zhao F, He H, Dai L, Yang J 2014 Effects of human disturbances on Korean pine coverage and age structure at a landscape scale in Northeast China. Ecol. Eng. 71, 375–379. (doi:10.1016/j.ecoleng.2014.07.072)

[58] Purugganan MD, Fuller DQ 2009 The nature of selection during plant domestication. Nature 457, 843–848. (doi:10.1038/nature07895) 19212403 10.1038/nature07895

[59] Speziale K, Lambertucci S, Gleiser G, Tella J, Hiraldo F, Aizen M 2018 Data from: An overlooked plant-parakeet mutualism counteracts human overharvesting on an endangered tree Dryad Digital Repository. (http://dx.doi.org/10.5061/dryad.9g263) 10.1098/rsos.171456 PMC5792925 29410848

